# Extra-mitochondrial mouse frataxin and its implications for mouse models of Friedreich’s ataxia

**DOI:** 10.1038/s41598-020-72884-w

**Published:** 2020-09-25

**Authors:** Liwei Weng, Laurent Laboureur, Qingqing Wang, Lili Guo, Peining Xu, Leah Gottlieb, David R. Lynch, Clementina Mesaros, Ian A. Blair

**Affiliations:** 1 Penn Medicine/CHOP Center of Excellence in Friedreich’s Ataxia, University of Pennsylvania, Philadelphia, PA 19104 USA; 2 Center of Excellence in Environmental Toxicology, Department of Systems Pharmacology and Translational Therapeutics, Perelman School of Medicine, University of Pennsylvania, Philadelphia, PA 19104 USA; 3 Departments of Pediatrics and Neurology, Perelman School of Medicine, University of Pennsylvania, Philadelphia, PA 19104 USA

**Keywords:** Isoenzymes, Multienzyme complexes, Neurodegenerative diseases

## Abstract

Mature frataxin is essential for the assembly of iron–sulfur cluster proteins including a number of mitochondrial enzymes. Reduced levels of mature frataxin (81-20) in human subjects caused by the genetic disease Friedreich’s ataxia results in decreased mitochondrial function, neurodegeneration, and cardiomyopathy. Numerous studies of mitochondrial dysfunction have been conducted using mouse models of frataxin deficiency. However, mouse frataxin that is reduced in these models, is assumed to be mature frataxin (78-207) by analogy with human mature frataxin (81-210). Using immunoaffinity purification coupled with liquid chromatography-high resolution tandem mass spectrometry, we have discovered that mature frataxin in mouse heart (77%), brain (86%), and liver (47%) is predominantly a 129-amino acid truncated mature frataxin (79-207) in which the N-terminal lysine residue has been lost. Mature mouse frataxin (78-207) only contributes 7–15% to the total frataxin protein present in mouse tissues. We have also found that truncated mature frataxin (79-207) is present primarily in the cytosol of mouse liver; whereas, frataxin (78-207) is primarily present in the mitochondria. These findings, which provide support for the role of extra-mitochondrial frataxin in the etiology of Friedreich’s ataxia, also have important implications for studies of mitochondrial dysfunction conducted in mouse models of frataxin deficiency.

## Introduction

**Figure 1:**
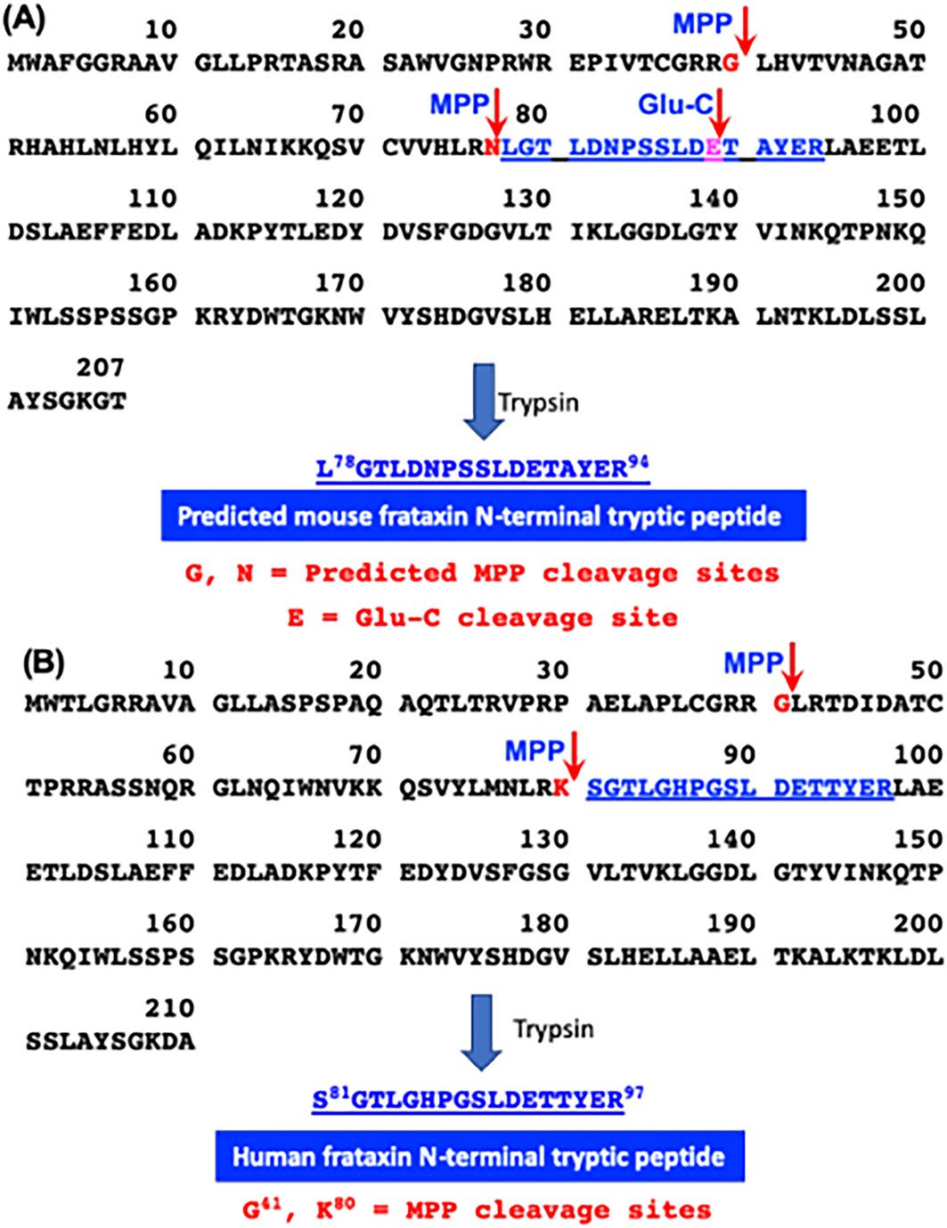
Amino acid sequences, predicted MPP cleavage sites, a Glu-C cleavage site, and N-terminal tryptic peptides. (**A**) Mouse mature frataxin (78-207) is formed by MPP-mediated cleavage of mouse full-length frataxin at G^40^-L^41^ followed by cleavage of the resulting intermediate frataxin (41-207) at N^77^-L^78^. Trypsin digestion of mature mouse frataxin results in formation of the N-terminal peptide L^78^GTLDNPSSLDETAYER^94^; whereas, digestion with the endopeptidase Glu-C results in formation of the N-terminal peptide L^78^GTLDNPSSLDETAYE^93^. (**B**) Human mature frataxin is formed by MPP-mediated cleavage of human full-length frataxin at G^41^-L^42^ followed by cleavage of the resulting intermediate frataxin (42-210) at K^80^-S^81^. Trypsin digestion of mature human frataxin results in formation of the N-terminal peptide S^81^GTLGHPGSLDE^89^.

The frataxin gene (*FXN*) encodes a highly conserved protein that is found in both prokaryotes and eukaryotes (Fig. 1)^1,2^. Frataxin is required for iron–sulfur cluster biogenesis^3,4^, and is involved in the formation of iron–sulfur cluster proteins that are localized to the mitochondrial compartment, such as lipoate synthase, aconitase, and succinate dehydrogenase^5^. Frataxin enhances sulfur transfer to the iron sulfur cluster assembly protein (ISCU), the scaffold on which iron–sulfur clusters are formed^6^, and accelerates the rate-limiting sulfur transfer step in the synthesis of [2Fe–2S] clusters^7^. Epigenetic silencing of the *FXN* gene^8^, results in a deficiency of frataxin protein and mitochondrial dysfunction^9^. Mitochondrial dysfunction resulting from frataxin deficiency is considered to be an underlying cause of the devastating genetic disease of Friedreich’s ataxia (FRDA)^10,11^, which is characterized by progressive neurodegeneration and hypertrophic cardiomyopathy^12,13^. In order to conduct mechanistic studies of mitochondrial dysfunction and/or to test potential therapeutic approaches for treating diseases such as FRDA, multiple mouse models of frataxin deficiency have been developed^14–22^.

Full length mouse frataxin (1-207) is expressed in the cytosol of all mouse cells and translocates to mitochondria where the mitochondrial targeting sequence is thought to be removed in two-step process by mitochondrial processing peptidase (MPP) analogous to that reported for human full-length frataxin (1-210)^23,24^. This would result in formation of a mouse mature mitochondrial form as the 130-amino acid mature frataxin (78-207, Fig. 1A) analogous to the 130-amino acid human mature frataxin (81-210, Fig. 1B)^23,24^. Mouse mature frataxin (78-207) has been characterized solely by analogy with the human protein and no structural studies have been performed on the protein that has been generated in vitro or in vivo. In addition, proteins with a leucine-residue at the amino terminus such as in mouse mature frataxin (78-207) are often converted to more stable forms, an observation known as the N-end rule^25^. This suggested that mature mouse frataxin was more heterogenous than human mature frataxin (81-210), which has a stable serine-residue at its amino terminus^23,24^. Furthermore, our discovery of an extra-mitochondrial form of human frataxin^26^ raised the possibility that there could be a corresponding extra-mitochondrial form of mouse mature frataxin. We have developed methodology based on immunoaffinity purification coupled with 2-dimensional ultra-high performance liquid chromatography-parallel reaction monitoring/mass spectrometry (IP 2D-nano-UHPLC-PRM/MS) for characterizing the different circulating proteoforms of mature frataxin in human subjects^26,27^. This methodology has now been applied to characterizing the major mature frataxin proteoforms that are present in mouse heart, brain, and liver.

## Results

### IP with an anti-frataxin mouse monoclonal antibody (mAb) efficiently isolates human mature frataxin

The LifeSpan Biosciences anti-frataxin mouse mAb (clone 1D9) cross-linked to protein G magnetic beads for IP isolation of mature frataxin prior to stable isotope dilution UHPLC-PRM/MS analysis^26,27^ is no longer available. Therefore, we tested whether two other Abs (Abcam mouse mAb 113691 and rabbit polyclonal antibody (pAb, 175402) could be used to isolate human and mouse mature frataxin from tissue homogenates. A human mature frataxin standard (25 ng) expressed through stable amino acid labeling by amino acids in cell culture (SILAC) containing a 6xHis tag^27^ was added to triplicate mouse heart tissue and IP conducted with protein G magnetic beads non-covalently bound to the anti-frataxin mouse mAb or the anti-frataxin rabbit pAb. IP with the rabbit pAb resulted in only 10.3% recovery of human mature SILAC-frataxin (81-201) (Supplementary Fig. 1A) when compared with IP using the mouse mAb (Supplementary Fig. 1B). An even lower recovery of mouse frataxin from mouse tissues with the rabbit pAb was observed (data not shown). The clear superiority of the mouse mAb for IP of both human and mouse mature frataxin proteoforms validated its use in all subsequent experiments.

### Mature frataxin is truncated in mouse heart, brain, and liver

**Figure 2:**
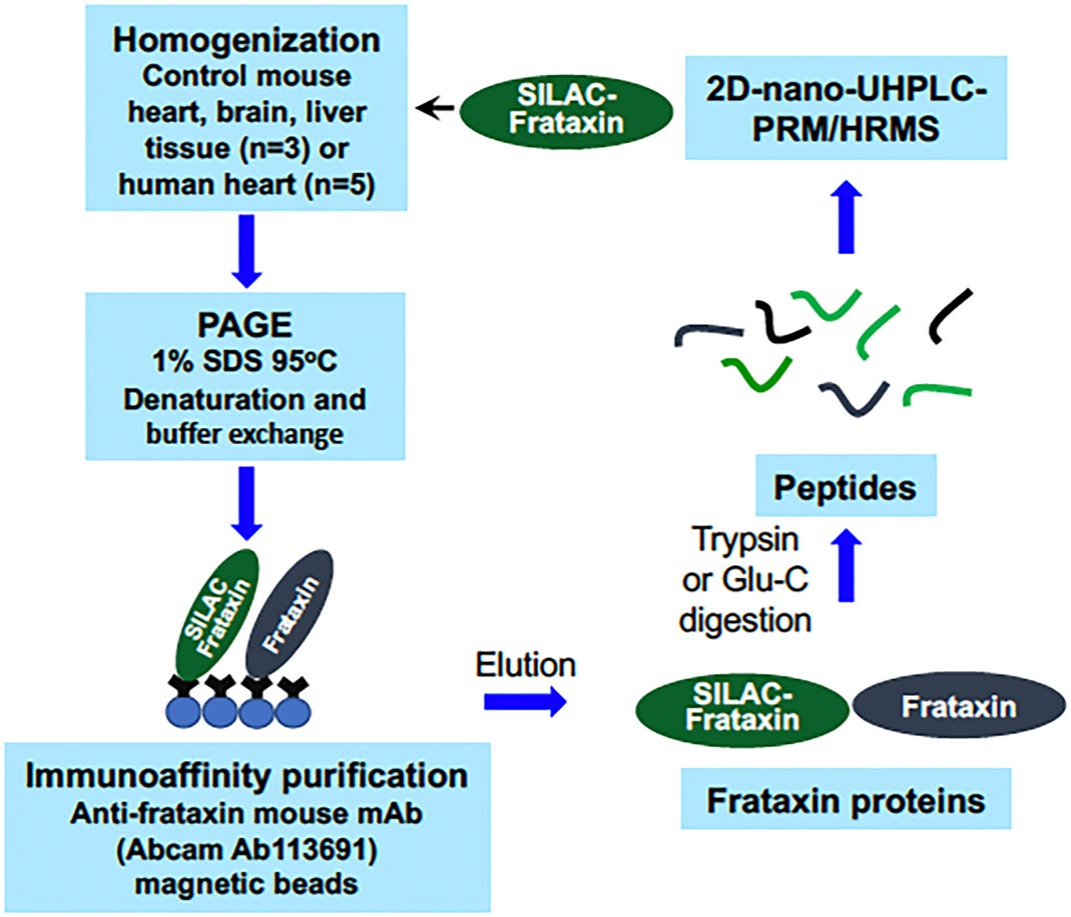
Scheme showing the protocol used for analysis of mature frataxin in mouse and human tissues.

**Figure 3:**
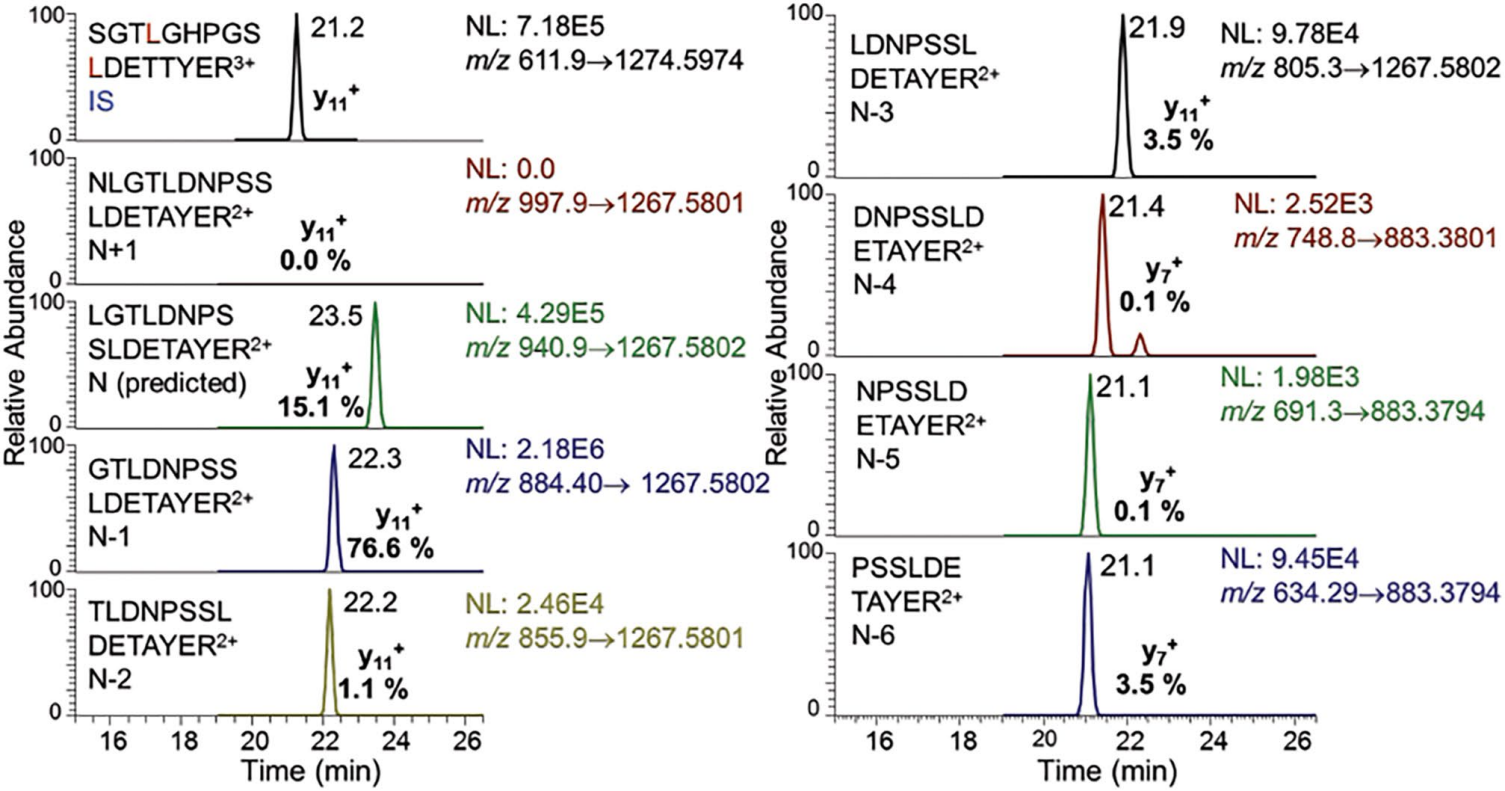
UHPLC-PRM/MS analysis of N-terminal tryptic peptides from mouse mature frataxin isolated from mouse heart tissue after addition of human SILAC-mature frataxin (81-210) internal standard.

**Figure 4:**
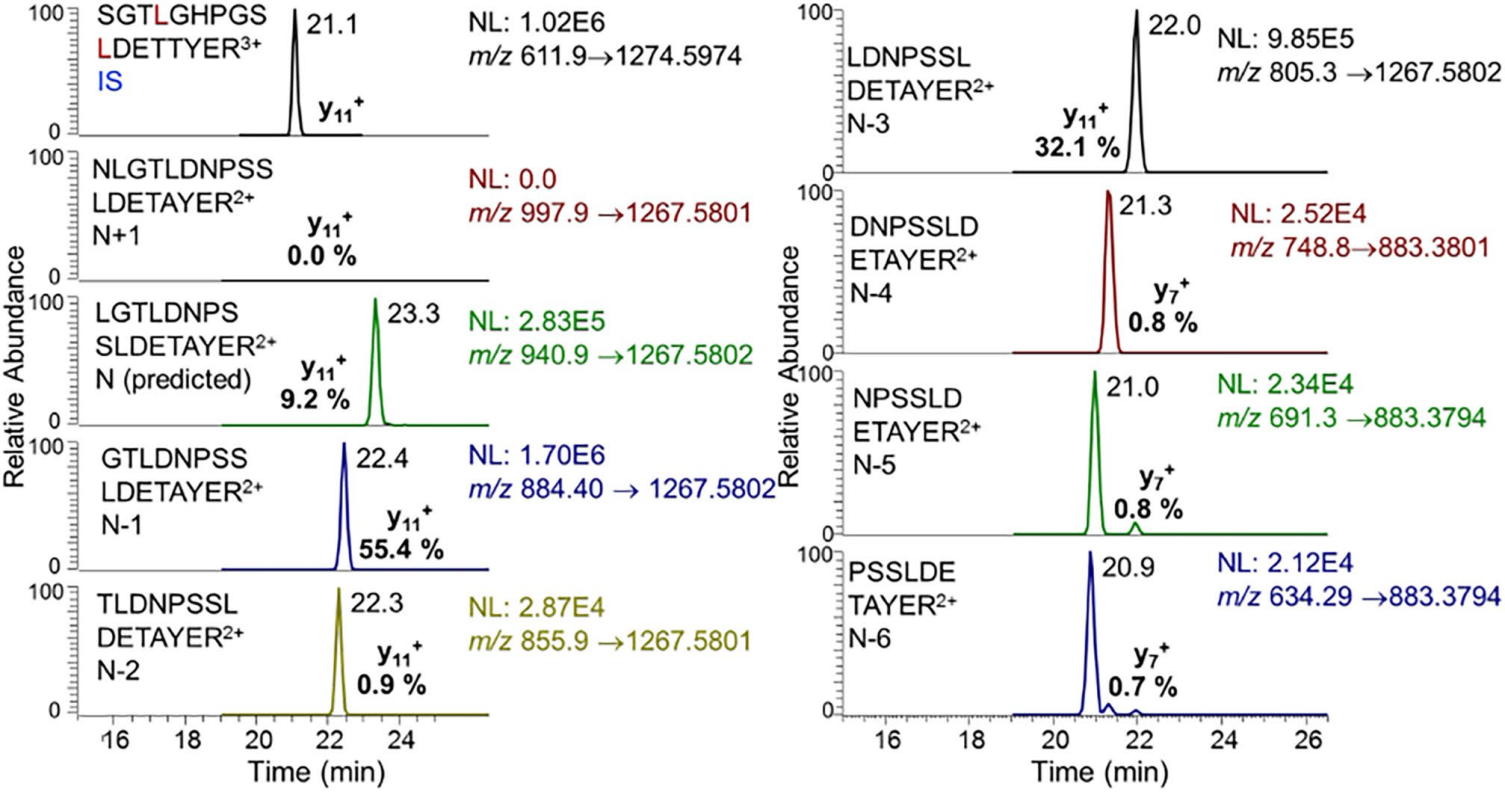
UHPLC-PRM/MS analysis of N-terminal tryptic peptides from mouse mature frataxin isolated from mouse liver tissue after addition of human mature SILAC-frataxin (81-210) internal standard.

Human mature SILAC-frataxin (81-210) was added to triplicate samples of mouse heart and brain tissue to act as an internal standard (Fig. 2). The SILAC-frataxin internal standard made it possible to assess whether any proteolysis occurred during isolation and IP of the frataxin and also allowed semi-quantitative analysis of mouse proteins to be conducted. Frataxin purified from the tissue samples was subjected to trypsin hydrolysis and the hydrolysate analyzed by 2D-nano-UHPLC-PRM/MS as described previously^27^. Potential N-terminal truncated tryptic peptides were subjected to in silico analysis using Protein Prospector to calculate the theoretical doubly- and triply-charged protonated molecules ([M + 2H]^2+^ and [M + 3H^3+^]). These ions were then employed for the UHPLC-PRM/MS analysis of tissue frataxin proteoforms. This resulted in the identification of one major (N-1) and two minor (N-3, N-6) truncated N-terminal tryptic peptides from mouse heart (Fig. 3) and brain (data not shown) with shorter UHPLC retention times than the predicted leucine-containing N-terminal mature mouse frataxin tryptic peptide (N, L^78^GTLDNPSSLDETAYER^94^, Fig. 1A). In addition, two major (N-1, N-3) and three minor (N-2, N-5, N-6) truncated N-terminal tryptic peptides were identified in mouse liver (Fig. 4). A second analysis conducted by UHPLC-tandem mass spectrometry (MS/MS) confirmed the identities of the predicted N-terminal peptide and the truncated peptides through the presence of diagnostic y-product ions. Thus, product ion spectra for the three major tryptic peptides corresponding to N (Supplementary Fig. 2A), and truncated peptides N-1 (Supplementary Fig. 2B), and N-3 (Supplementary Fig. 2C) had almost identical y-ions (from y_4_^+^ to y_12_^+^) although they had different relative abundances because they were generated from different doubly charged parent ions. The three minor N-terminal truncated tryptic peptides (N-2, N-5, N-6) had the predicted product ion spectra (data not shown). All of the other major tryptic peptides from mouse mature frataxin (78-207) including mouse specific-peptide N^168^WVYSHDGVSLHELLAR^185^ and mouse/human common-peptides L^133^DLSSLAYSGK^144^, Q^150^IWLSSPSSGPKR^161^, and L^195^DLSSLAYSGK^205^, were characterized by their product ion spectra and retention times (data not shown).

### Truncated proteins are the major mature frataxin proteoforms in mouse tissues

**Figure 5:**
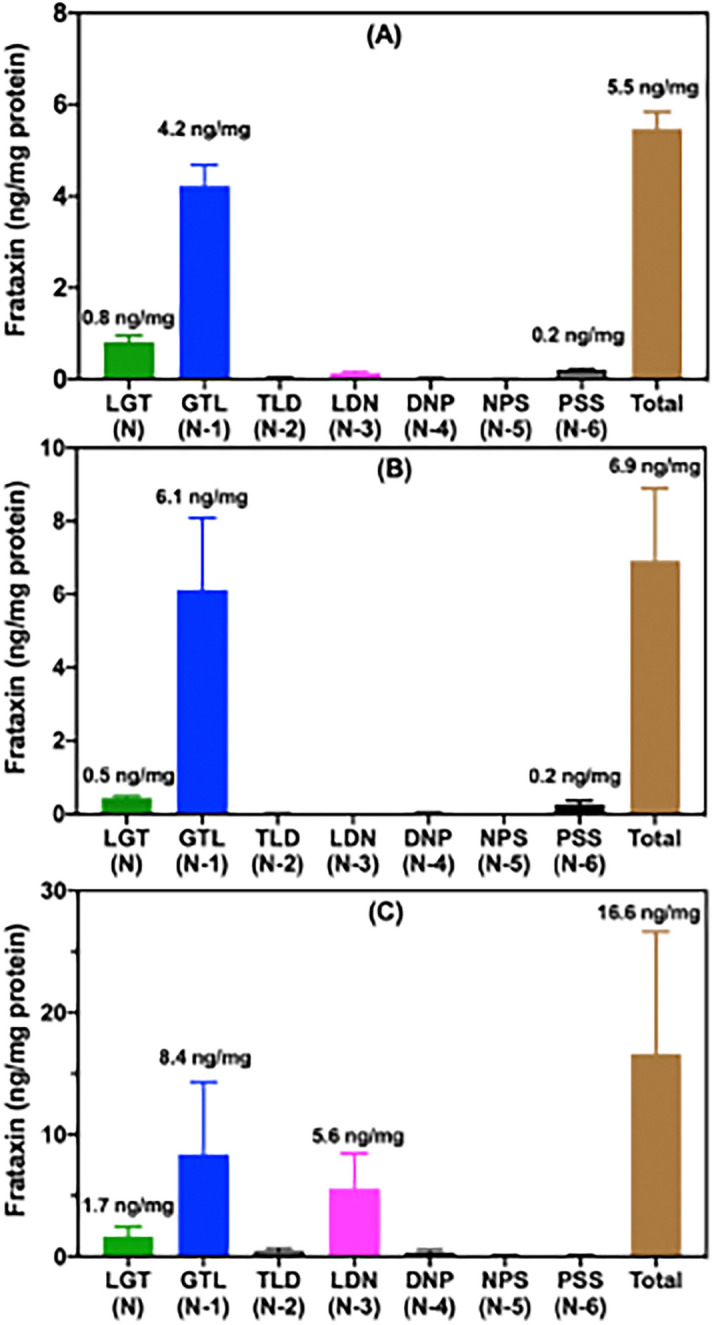
Relative amounts of mouse frataxin proteoforms in mouse tissues. (**a**) Heart (n = 3). (**b**) Brain (n = 3). (**c**) Liver (n = 3).

Authentic samples of pure mouse mature frataxin (78-207) and the truncated versions discovered in the present study are not available. Therefore, semi-quantitative analysis was conducted by comparison of their UHPLC-PRM/MS intensities with the signal from N-terminal tryptic peptide of purified human mature SILAC-frataxin (81-210), which is 98.5% homologous, and had been carried through the same IP isolation procedure (Fig. 2). The amounts of total frataxin [mature frataxin (78-207) and truncated forms] in mouse heart, brain, and liver were 5.5 ng/mg tissue (Fig. 5A), 6.9 ng/mg tissue (Fig. 5B) and 16.6 ng/mg tissue (Fig. 5C), respectively. The major proteoform characterized in all three tissues was the N-1 truncated mature frataxin (79-207), which accounted for 77.2%, 86.9%, and 47.0% of the mature frataxin present in mouse heart, brain, and liver, respectively (Fig. 5, Table 1). Mouse mature frataxin (78-207, Fig. 1A) predicted by analogy with human mature frataxin (81-210, Fig. 1B) only accounted for 14.9%, 7.3%, and 11.3% of the frataxin present in mouse heart, brain, and liver, respectively (Fig. 5, Table 1). A second major truncated mature frataxin proteoform (N-3, 81-207) was identified in mouse liver, corresponding to 5.6 ng/mg tissue (Fig. 5), which accounted for an additional 36.0% of the liver frataxin (Table 1). There was a minor truncated mature frataxin proteoform in mouse heart and brain (N-6, 83-207), which accounted for 4.5% of the total frataxin (Fig. 5, Table 1). A trace amount (< 1%) of the major truncated liver mature frataxin proteoform (N-3, 81-207) was found in the heart but none was identified in the brain (Fig. 5, Table 1). Three minor truncated proteoforms were detected in mouse liver (N-2, N-5, N-6) each corresponding to < 3% of the total liver mature frataxin (Fig. 5, Table 1). The mature frataxin proteoform corresponding to N-4 (82-207) or any truncations longer that N-6 were not detected in any of the mouse tissues.

**Table 1:** Relative amounts of individual mature frataxin proteoforms in mouse tissues (n = 3).

Tissue	LGT (N)		N-1 (GTL)		N-2 (TLD)		N-3 (LDN)		N-4 (DNP)		N-5 (NPS)		N-6 (PSS)	
	%	SEM	%	SEM	%	SEM	%	SEM	%	SEM	%	SEM	%	SEM
Heart	14.9	2.9	77.2	9.3	0.7	0.2	2.7	0.8	0.5	0.2	0.2	0.1	3.8	0.8
Brain	7.3	1.7	86.9	3.4	0.3	0.2	0.1	0.1	0.7	0.3	0.1	0.1	4.6	2.6
Liver	11.3	2.5	47.0	8.4	2.8	1.2	36.0	4.6	1.8	0.4	0.5	0.1	0.6	0.1

### Truncated frataxin is the major mature frataxin proteoform in mouse liver cytosol

Mouse liver was fractionated in the presence of human mature SILAC-frataxin (81-210) into nuclei, mitochondria, and cytosol. There was undetectable contamination of the nuclear and mitochondrial fractions by cytosol as evidenced by the lack of FASN protein (Supplementary Fig. 3A, lanes 1–4); undetectable contamination of the cytosolic fractions by intact mitochondria as evidenced by the absence of VDAC protein Supplementary Fig. 3B, lanes 5 & 6) but some contamination of the nuclear fraction revealed by the presence of the VDAC protein (Supplementary Fig. 3B, lanes 1 & 2); and undetectable contamination of cytosol or mitochondrial fractions by nuclei as shown by the absence of histone H3 and H4 (Supplementary Fig. 3C, lanes 3–6). The presence of citrate synthase in each fractions (data not shown) indicated that some mitochondria had been disrupted during the liver tissue freeze/thaw process. Therefore, there was some contamination of the cytosolic and nuclear fraction by the released mitochondrial proteins. The major frataxin protein observed in the cytosol was the N-1 truncated mature frataxin (79-207) proteoform based on analysis of its N-terminal frataxin tryptic peptide. Truncated mature frataxin (79-207) accounted for 96% of the cytosolic frataxin with the remaining 4% present as the predicted mitochondrial form of the protein, mature frataxin (78-207) (Table 2). This proteoform most likely arose in the cytosol through contamination by disrupted mitochondria during freeze thawing. Full-length mouse frataxin (1-207) and intermediate mouse frataxin (41-207) were not detected in the cytosolic fraction. Mouse mature frataxin (78-207) was much more enriched in mitochondrial and nuclear fractions compared with the cytosolic fraction comprising 36.4% and 29.8%, respectively of the mature frataxin that was found (Table 2). Unfortunately, the other major mouse mature frataxin proteoform (N-3; 81-207) identified in mouse liver was lost during the sub-cellular fractionation protocol that was conducted in duplicate on three separate occasions. Conversely, excellent recoveries of human mature SILAC-frataxin (81-201), mouse mature frataxin (78-207; N), and mouse truncated mature frataxin (79-207; N-1) were obtained each time. This suggests that the human SILAC frataxin standard was able to act as a carrier to prevent any losses of mature frataxin (78-207; N) and mouse truncated mature frataxin (79-207; N-1) but did not act as a carrier for mouse mature frataxin (N-3; 81-207). In a previous study of amyloid peptides, we observed significant losses in the absence of a stable isotope standard carrier due to non-covalent binding to the glassware^28^. Therefore, we suspect that mouse truncated mature frataxin (81-210; N-3) was lost in a similar manner and that the availability of a SILAC form of the frataxin protein would prevent this loss.

**Table 2:** Sub-cellular localization of individual mature frataxin proteoforms in mouse liver (n = 2).

Fratax in proteoform localization	LGT (N) amount		N-1 (GTL) amount		Total frataxin (ng/mg tissue)	Ratio GTL/LGT
	ng/mg protein	%	ng/mg protein	%		
Nucleus	1.4	29.8	3.3	70.2	4.7	2.4
Mitochondria	0.4	36.4	0.7	36.6	1.1	1.8
Cytosol	0.5	4.0	11.9	96.0	12.4	23.8

### Truncation of mouse frataxin does not occur during isolation and analysis

As a control for potential proteolysis during the analysis of mouse tissues, human frataxin carried through the isolation and analysis procedure was analyzed to determine if any truncated N-terminal peptides were formed. Retention times of the expected N-terminal peptide (N) and the six potential N-terminal truncated peptides were determined from synthetic unlabeled peptides obtained commercially from New England Peptides (Supplementary Fig. 4 red arrows). The only frataxin-related PRM/MS signal in each of the mouse tissue samples arose from intact labeled N-terminal tryptic peptide (S^81^GT**L**GHPGS**L**DETTYER^97^), which eluted at a retention time of the authentic peptide (21.2 min, Supplementary Fig. 4) and exhibited all of the expected product ions (data not shown). This showed that no proteolysis of frataxin occurred during isolation and analysis of the mouse tissue samples.

### Concentration of mature frataxin (81-210) in human heart tissue

**Figure 6:**
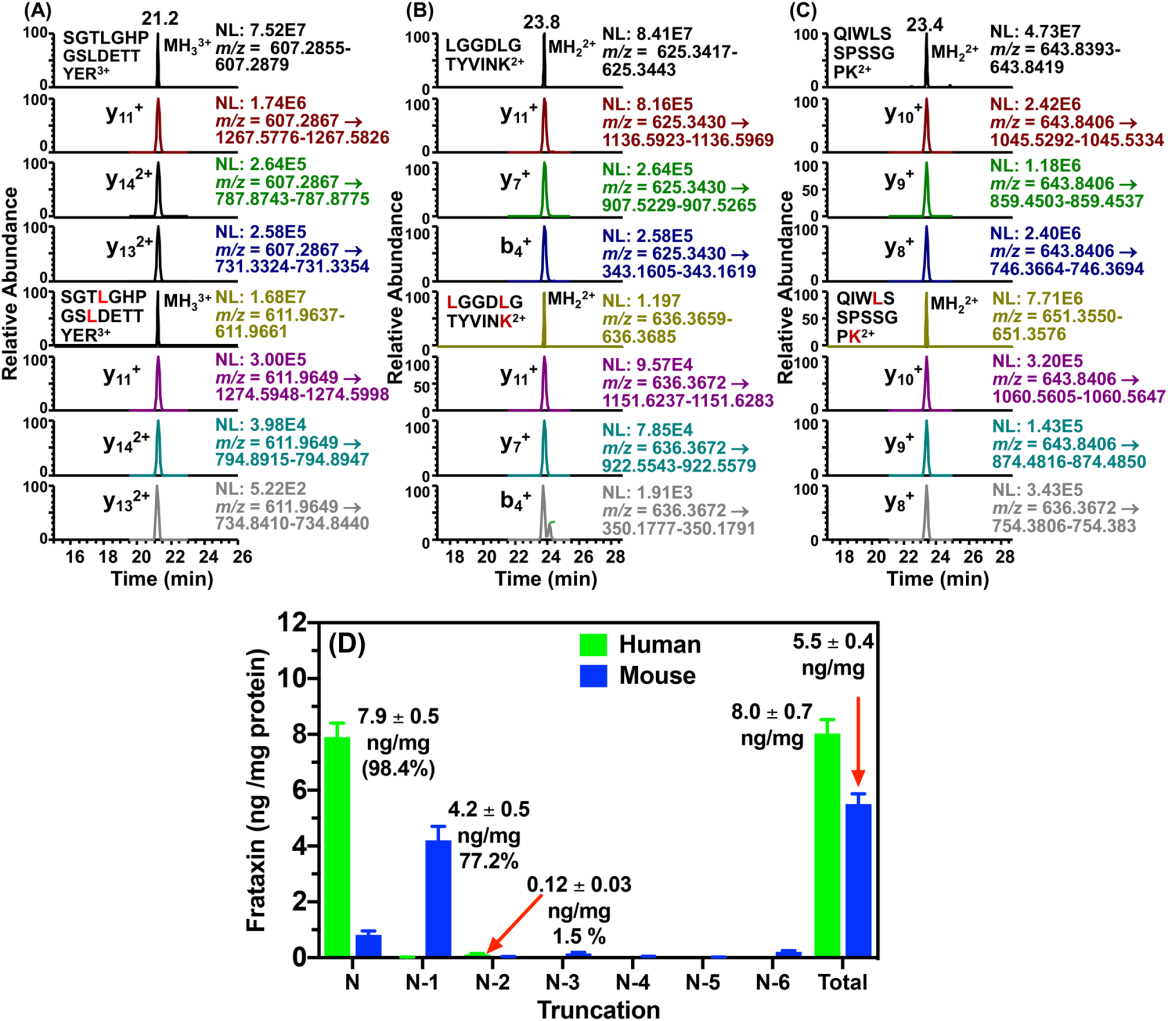
LC-PRM/MS analysis of human heart mature frataxin and human mature SILAC-frataxin (81-210) tryptic peptides (**A**). S^81^GTLGHPGSLDETTYER^97^ and S^81^GT**L**GHPGS**L**DETTYER^97^. (**B**) L^136^GGDLGTYVINK^147^ and **L**^**136**^GGDLGTYVIN**K**^**147**^. (**C**) Q^150^IWLSSPSSGPK^161^ and Q^150^IW**L**SSPSSGP**K**^**161**^. (**D**) Relative amounts of frataxin proteoforms in human heart (n = 5) and mouse heart (n = 3). **L** = [^13^C_6_^15^N_1_]-leucine, **K** = [^13^C_6_^15^N_2_]-lysine. PRM/MS ions used for quantification of tryptic peptides were: S^81^GTLGHPGSLDETTYER^97^ y_11_^+^, y_14_^2+^, and y_13_^2+^; L^136^GGDLGTYVINK^147^, y_11_^+^, y_7_^+^, and b_4_^+^; Q^150^IWLSSPSSGPK^161^ y_10_^+^, y_9_^+^, and y_8_^+^. Accurate masses that were monitored are shown on the chromatograms.

Mature frataxin was isolated from frozen non-failing human heart tissue obtained from five different subjects. The frataxin was extracted from homogenized tissue by IP in the presence of human mature SILAC-frataxin (81-210) (Fig. 2). Using trypsin digestion in combination with our validated 2D-nano-UHPLC-PRM/MS assay^27^, the mature frataxin concentrations were then determined (Figs. 6A–C). The concentration of mature frataxin (81-210) was 7.9 ± 0.5 ng/mg tissue, which was similar to that determined for the total mature frataxin proteoforms in control mouse heart tissue (5.5 ± 0.4 ng/mg tissue, Fig. 6D). However, in contrast to the mouse tissue, there was remarkable concordance of the N-terminal tryptic peptide [S^81^GTLGHPGSLDETTYER^97^, Fig. 6A) with the other tryptic peptides (L^136^GGDLGTYVINK^147^, Fig. 6B; Q^150^IWLSSPSSGPK^161^, Fig. 6C) used for the quantification for all five of the human heart samples. The mature frataxin isolated by IP from human heart samples (n = 5, Fig. 2) was also analyzed by 2D-nano-UHPLC-PRM/MS using parent ions for the predicted intact N-terminal peptide together with parent ions calculated by Protein Prospector (https://prospector.ucsf.edu/prospector/mshome.htm) for peptides with truncations from N-1 to N-6 (Supplementary Fig. 5). Retention times of the expected N-terminal peptide (N) and the six potential N-terminal truncated peptides (N-1 to N-6) were determined from synthetic peptides obtained commercially from New England Peptides (Supplementary Fig. 5, red arrows). The major frataxin-related PRM/MS signal in each of the five human heart samples arose from intact N-terminal tryptic peptide (S^81^GTLGHPGSLDETTYER^97^), which corresponded to 98.4% (7.9 ng/mg protein) of the mature frataxin (Supplementary Fig. 5), eluted at a retention time of the authentic peptide (21.3 min, Supplementary Fig. 5) and exhibited all of the expected product ions (data not shown). Weak signals from N-terminal truncated peptides were observed at the retention times corresponding to G^82^TLGHPGSLDETTYER^97^ (N-1, 0.01 ng/mg tissue; 0.1%) and T^83^LGHPGSLDETTYER^97^ (N-2, 0.12 ng/mg tissue; 1.5%) were observed (Fig. 6D and Supplementary Fig. 5). This showed that frataxin in human heart was present primarily as mature frataxin (81-210), that it underwent minimal proteolysis i*n vivo*, and that it could be isolated with minimal proteolytic degradation.

### Mouse mature frataxin is not elongated at the amino terminus

We have previously identified a human mature frataxin extra-mitochondrial proteoform (isoform E) with a five-amino acid elongation at the amino terminus^26^. This raised the possibility that an elongated proteoform of mature frataxin could be present in mouse tissues. The presence of R^75^ in mouse frataxin (Fig. 1A) prevents the formation of tryptic peptide from full-length frataxin containing an analogous number of amino acids to the human protein (Fig. 1B). This problem was overcome in the human protein by the use of Asp-N-digestion at the N-terminal side of D^91^ to generate a 15-amino acid peptide^26^. However, mouse frataxin contains D^82^ (Fig. 1A), which is not present at an equivalent position in human frataxin (Fig. 1B). This prevented a similar strategy from being used because very short peptides would have been generated. Therefore, a Glu-C digestion of the frataxin isolated from mouse tissues was used and the N-terminal peptides arising from mouse tissue frataxin through cleavage at the carboxy-terminal-side of E^89^ (Fig. 1A) were analyzed by UHPLC-PRM/MS (Supplementary Fig. 6). No elongated peptides were observed for mouse heart, brain, or liver frataxin. However, Glu-C peptides corresponding to the four major N-terminal peptides that had been identified from the trypsin digest were observed. The full length (N) form (L^78^GTLDNPSSLDE^89^), N-1 (G^79^TLDNPSSLDE^89^), N-2 (T^90^LDNPSSLDE^89^) and N-3 (L^81^DNPSSLDE^89^) were observed in ratios similar to those observed for the corresponding tryptic peptides (Supplementary Fig. 6, Table 1). No frataxin-related N-terminal acetylated Glu-C peptides were identified in any of the mouse tissue samples.

## Discussion

A mouse analog of the human isoform E is not expressed in mouse tissues because mouse *FXN* (Fig. 1A) lacks the alternative splice site start codon for M76 found in exon-2 of human *FXN* (Fig. 1B). Therefore, other mature mouse frataxin proteoforms would likely arise from either the truncation of the mature mitochondrial frataxin or by proteolysis of the full-length cytosolic frataxin as we had observed for isoform E in human HEK cells^26^. We have never detected full-length human frataxin or full-length mouse frataxin in tissue samples either by western blot or by mass spectrometry. As far as we are aware, the only instance of endogenous full-length frataxin detection is as a result of its over-expression in cell culture. In addition, we did not detect any full-length frataxin protein in either the human or mouse tissue samples that were analyzed in the present study. Therefore, a SILAC mature human frataxin standard (rather than a SILAC full-length human frataxin) was added to the mouse tissue homogenates to serve as an internal standard and to provide a control to determine whether any proteolysis of the frataxin occurred over the course of the IP from tissue homogenates (Fig. 2). To date, a non-specific protease capable of truncating frataxin in mammalian cells has not yet been identified. However, legumain, an asparaginyl peptidase recently identified in macrophages, can hydrolyze the full-length mouse frataxin at arginine-77^29^. Human mature SILAC-frataxin (81-210) is 98.5% homologous and 92.3% identical with the predicted sequence of mouse mature frataxin (78-207). Proteolysis of mature SILAC-frataxin (81-210) would be readily apparent on UHPLC-MS/MS analysis of the N-terminal tryptic peptide if any had occurred. This revealed that no proteolysis had occurred during the analytical procedure because only product ions from [M + 3H]^3+^ (including the intense y_11_^+^-ion) from the N-terminal tryptic-peptide S^81^GT**L**GHPGS**L**DETTYER^97^ were detected (Fig. 4 and Supplementary Fig. 4; **L** = [^13^C_6_^15^N_1_]-leucine). In contrast, product ions from [M + 2H]^2+^ (including the intense y_11_^+^-ion) of the predicted tryptic-peptide L^78^GTLDNPSSLDETAYER^94^ from mouse mature frataxin (78-207) were present in mouse heart frataxin together with product ions derived from the [M + 2H]^2+^ of three truncated forms corresponding to N-1, N-3, and N-6 (Fig. 3). Specificity of mouse MPP for the R-2 site^30^ was confirmed by the absence of the tryptic peptide N^77^LGTLDNPSSLDETAYER^94^, which would have arisen from cleavage between R^76^-N^77^ (Figs. 3 and 4). Similar results to mouse heart were obtained when the tryptic peptides from mouse brain frataxin was analyzed except that only two of the truncated tryptic-peptides (N-1 and N-6) were detectable (Table 1). In contrast, the N-3 tryptic peptide from mouse liver frataxin was found in similar amounts to the N-1 tryptic peptide together with two additional minor truncated peptides corresponding to N-2 and N-4 (Fig. 4). Additional studies with Glu-C digestion, confirmed that there were no elongated N-terminal peptides in mouse tissue. This is exemplified for mouse liver frataxin by the absence of any elongated peptides (Supplementary Fig. 6). Furthermore, no N-terminally acetylated Glu-C peptides were detected. However, truncated N-terminal Glu-C peptides were observed (Supplementary Fig. 6) in similar ratios to those observed for the corresponding truncated N-terminal tryptic peptides (Table 1).

**Figure 7:**
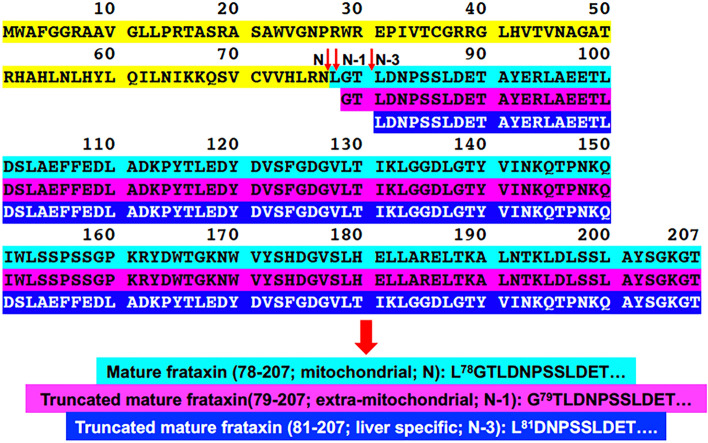
Amino acid sequences of newly identified mouse mature frataxin proteoforms. Peptides containing amino acids 1–77 that are lost through MPP-mediated cleavage of full-length frataxin (1-207) are highlighted in yellow. Sequences of mature frataxin (78-207; N), truncated mature frataxin (79-207; N-1), and truncated mature frataxin (81-207; N-3) are highlighted in cyan, purple, and blue, respectively.

Semi-quantitative analysis of mouse tissue frataxin was conducted by comparison of the UHPLC-PRM/MS signals from the predicted (N) and truncated (N-1 to N-6) mouse frataxin tryptic peptides to the signal from the human mature SILAC-frataxin (81-210) SGT tryptic peptide derived from 25 ng of human mature SILAC-frataxin (81-210) (Fig. 5). This revealed that the mouse mitochondrial mature frataxin (78-207) predicted by analogy with the human protein (Uniprot O35943) only accounted for 14.9%, 7.3%, and 11.3% of the mature frataxin present in mouse heart, brain, and liver, respectively (Table 1). The major truncated mature frataxin (79-207) in mouse heart and brain corresponds to the loss of the N-terminal leucine residue leaving an N-terminus of G^79^TL rather than L^78^GT (Fig. 7). The N-6 truncation to give mature frataxin (84-207) in heart and brain corresponds to approximately 4% of the total frataxin (Table 1). The two major proteoforms in mouse liver corresponding to N-1 (GTL, 47.0%) and N-3 (LDN, 36.0%) accounts for 83% of the total mature frataxin in mouse liver (Table 1). Interestingly, the truncated LDN-peptide from mature frataxin (81-207) found in mouse liver was barely detected in mouse heart and was not detected in mouse brain indicating there might be an additional role for this proteoform in mouse liver. The three minor proteoforms in mouse liver corresponding to N-2, N-5, and N-6 truncations were present at levels of < 3% of the total mature frataxin (Table 1).

Although we were able to show that the truncations in mouse mature frataxin did not result from tissue extraction and IP, it was conceivable that proteolysis had occurred during the harvesting of tissues from the mice before they were frozen in liquid nitrogen. In order to address this possibility, mature frataxin was analyzed in samples of human heart (n = 5) that were obtained in a similar way to the mouse tissues. The ratio of the intensity of the intact N-terminal peptide (S^81^GTLGHPGSLDETTYER^98^) to its corresponding SILAC-internal standard peptide was similar to the ratios of other tryptic frataxin peptides such as L^136^GGDLGTYVINK^147^ and Q^153^IWLSSPSSGPK^164^ to their human mature SILAC-frataxin internal standards (Fig. 6A)^27^. Weak signals for N-terminal truncated peptides from human mature frataxin (81-210) were observed at the retention times corresponding to G^82^TLGHPGSLDETTYER^97^ (N-1, 0.01 ng/mg tissue, 0.1%) and T^83^LGHPGSLDETTYER^97^ (N-2, 0.15 ng/mg tissue, 1.5%; Fig. 6 and Supplementary Fig. 5). The major signal arose from S^81^GTLGHPGSLDETTYER^97^ (N, 7.9 ng/mg tissue, 98.4%; Fig. 6D and Supplementary Fig. 5), which showed that mature frataxin (81-210) could be isolated from human heart tissue with minimal proteolytic degradation. As very little proteolysis of mature frataxin (81-210) was found to occur during the harvesting of human heart and none occurred during extraction and IP of the protein, which provides compelling evidence that the truncation of mature frataxin (78-207) in mouse tissues occurs primarily in vivo. This means that the major frataxin proteoform in the mouse is the 129-amino acid truncated mature frataxin (79-207, Fig. 7) and not the 130-amino acid form of mature frataxin (78-207) annotated by Uniprot O35943 through similarity to the human protein (Fig. 7). There is another major truncated (127-amino acid) frataxin protein in mouse liver—truncated mature frataxin (81-207) (Fig. 7).

The ratio of extra-mitochondrial truncated mature frataxin (79-207) to mitochondrial mature frataxin (78-207) in the mitochondrial fraction from mouse liver is 1.8 compared with the cytosolic fraction where the ratio is 24.3 (Table 2). The relatively small amount of extra-mitochondrial truncated mature frataxin (79-207) found in the mitochondrial fraction is thought to result from the trace amount of cytosol (approximately 4%) that was not removed during isolation of the mitochondria. In contrast, mitochondrial mature frataxin (78-207) found in the cytosolic fraction is thought to arise from disruption of the mitochondria and secretion of soluble proteins into the cytosol during freeze/thawing. It is difficult to prevent contamination of the nuclear fraction with mitochondria as evidenced by the presence the VDAC mitochondrial membrane protein (Supplementary Fig. 3B). This resulted in a ratio of extra-mitochondrial truncated mature frataxin (79-207) to mitochondrial mature frataxin of 2.4 (Table 2). It seems likely that extra-mitochondrial truncated mature frataxin (79-207) characterized in the present study was not recognized by the mAb used to detect cytosolic frataxin in an earlier study^17^. Initially, we had considered conducting the subcellular fractionation studies using fresh mouse heart, brain, and liver. Ultimately, we elected to use frozen tissues for three reasons. First, and perhaps most importantly, we wanted to ensure that any possible proteolysis was minimized by rapidly freezing the tissues in liquid nitrogen. Second, we wanted our work to be consistent with previous studies so that we could draw comparisons between our findings and other studies of mouse frataxin proteoforms^14,19,21^ and their subcellular localization^17^. Finally, our goal was to use a method that could be applied to human tissues. Since it would be impossible to use fresh human tissues, we were limited to the use of frozen tissue.

It is well known that mitochondria undergo some disruption during freeze-thawing. However, it would have required > 95% of the mitochondria to have been disrupted in order to observe truncated mature frataxin (79-207) in the cytosolic fraction if it had actually been present in the mitochondria at the time of collection. The method for subcellular fractionation has been used in many studies to obtain mitochondria^17,31^. Furthermore, we consistently saw less mature frataxin (78-207) in the brain (7.3 ± 1.7%) than in both liver and heart as well as less mature frataxin (78-207) in the liver (11.3 ± 2.5%) than in the heart (14 ± 2.9%) (Table 1). Therefore, it is highly unlikely that > 95% of the mitochondria were disrupted during the freeze-thawing process or that mitochondria were protected during freeze-thawing of the heart compared with the liver or brain. Mouse mature frataxin (79-207) could play an important role as extra-mitochondrial frataxin in the etiology of FRDA, as has been suggested previously^17,32–35^. More rigorous mass spectrometry-based studies with authentic labeled and unlabeled truncated mature mouse frataxin protein standards will be required to fully address this possibility. The second truncated form of mature frataxin (81-207) identified in mouse liver was lost during the sub-cellular fractionation process. A mouse mature SILAC-frataxin (81-210) standard would provide a carrier so that the endogenous form is not lost during isolation.

The presence of a leucine residue at the amino-terminus of mitochondrial mouse frataxin (L78GT) renders it unstable according to the N-end rule^25^. Typically, such proteins are modified through the action of intermediate cleaving peptidases (ICPs)^36^ such as XPNPEP^37^, which is present in both the mitochondria and the cytosol of mammalian cells. XPNPEP3 sequentially cleaves individual residues as it has been observed for the mitochondrial cysteine desulfurase, NFS1^37^. It is known that in human HEK cells, the chaperone protein GRP75 can increase frataxin levels perhaps by binding to frataxin reducing its degradation^38^. This would then suggest that the binding of GRP75 to mitochondrial mature mouse frataxin (78-207), which is a result of MPP-catalyzed proteolysis of the full-length mouse frataxin protein, could account for mature frataxin stabilization in mitochondria. This interaction would then limit ICP-mediated degradation. We have previously shown that frataxin isoform E undergoes proteolysis to form the mature human frataxin (81-210) in the cytosol of HeLa cells^26^. Therefore, it is conceivable that other frataxin proteoforms can be processed in mammalian cells without requiring the action of mitochondrial MPP. In fact, the asparaginyl endopeptidase, legumain, was shown recently to cleave full-length mouse frataxin at asparagine-77, producing mature mouse frataxin (78-207)^29^. Without the protection of mitochondrial GRP75, cytosolic mature mouse frataxin could then be a substrate for XPNPEP3 action. Removal of this N-terminal leucine residue would then generate truncated mature mouse frataxin (79-207)^29^, the major proteoform that we found in mouse tissues. Subsequent sequential hydrolysis of single N-terminal residues would then result in additional truncated proteoforms. Intriguingly, the N-3 protein identified in mouse liver has an N-terminal leucine residue (L^81^DP), suggesting the presence of a liver-specific chaperone that plays a role in protecting this N-3 proteoform.

In light of our findings, it will be necessary in future studies with mouse models to define the specificity of antibodies for each of the three major proteoforms that have been identified (Fig. 7). It will also be important to determine which mature frataxin proteoforms have been modulated and to establish how down-regulation of each of the individual proteoforms contributes the observed neurodegeneration and cardiotoxicity. The characterization of an extra-mitochondrial frataxin proteoform will have substantial implications for a role in the etiology and treatment of FRDA. Extra-mitochondrial frataxin could be involved in assembly of extra-mitochondrial iron-sulfur cluster proteins such as DNA repair enzymes^39^ and helicases involved in telomere length control^40^, which are known to be dysregulated in FRDA. However, it is conceivable that the mitochondrial mature frataxin (81-210) in humans is also present in the cytosol because, unlike the corresponding mouse proteoform, it lacks an N-terminal leucine residue and so is quite stable. Apart from our own studies on frataxin isoform E, several other studies have suggested that human mature frataxin can have an extra-mitochondria location. Alternatively, processing of the mature mouse frataxin could proceed in a different manner than in humans. If this is the case, we would suggest that mouse models do not serve as a good model for humans. Finally, as human gene therapy is tested in mouse models, it is possible that the mature human protein will undergo truncations in the mouse tissues, although they will most likely be at different sites because of the differences in amino acid sequence at the amino-terminus (Fig. 1B) compared to mouse frataxin (Fig. 1A). This will impact on the assessment of efficacy and safety of the human transgene constructs (such as CAG-h*FXN*-HA) in mouse models^19,21^.

## Methods

### Reagents and supplies

All reagents and solvents were of LC–MS grade quality unless otherwise noted.[^13^C_6_^15^N_2_]-lysine and [^13^C_6_^15^N_1_]-leucine were from Cambridge Isotope Laboratories (Andover, MA, USA). Both anti-frataxin mouse mAb (ab113691) for immunoprecipitation and western blot and anti-frataxin rabbit pAb (ab175402) were purchased from Abcam (Cambridge, MA). The anti-histone H4 rabbit pAb (cat. # 16047-1-AP) was purchased from Proteintech (Rosemont, IL). Anti-histone H3 rabbit pAb (cat. # PA5-16183) and anti-fatty acid synthase (FASN) rabbit pAb (cat. # MA5-14887) were purchased from Invitrogen (Carlsbad, CA). Anti-voltage dependent anion channel protein (VDAC) rabbit mAb (clone D73D12, cat # 4661) was from Cell Signaling Technology (Danvers, MA). An IRDye 800CW Goat anti-Rabbit IgG Secondary Antibody (#926-32211) was purchased from LI-COR (Lincoln, NE). Trypsin protease was obtained from Promega (Madison, WI). EDTA-free protease inhibitor cocktail, bovine serum albumin (BSA), DL-dithiothreitol (DTT), β-mercaptoethanol (BME), and Glu-C V8 protease, were purchased from MilliporeSigma (Billerica, MA). LC grade water and acetonitrile were from Burdick and Jackson (Muskegon, MI, USA). Corning Dulbecco’s Phosphate-Buffered Saline, 1X without calcium and magnesium, Pierce protein G magnetic beads, Tween 20, NuPAGE 12% Bis–Tris protein gels, NuPAGE LDS sample buffer, and colloidal Coomassie blue staining kit were obtained from ThermoFisher Scientific (Grand Island, NY). Amicon Ultracel-50K filters, and Eppendorf LoBind microcentrifuge tubes were purchased from MilliporeSigma. Bradford protein assay reagent was obtained from Bio-Rad (Hercules, CA). Synthetic peptide standards were obtained from New England Peptide (Gardner, MA 01440).

### Tissue sample homogenate

Mouse heart, liver and brain samples from WT C57BL/6 mouse were snap-frozen in liquid nitrogen and stored at − 80 °C. They were provided by Dr. Wellen (Associate Professor of Cancer Biology, Perelman School of Medicine, University of Pennsylvania). Deidentified whole human hearts were procured from nonfailing brain dead organ donors with no history of heart failure were obtained at the time of orthotopic heart transplantation as part of the Hospital of the University of Pennsylvania (HUP) donation and biobanking program. All hearts received in situ cold cardioplegia and were placed on wet ice in 4 °C Krebs–Henseleit buffer. Five transmural left ventricular samples, excluding epicardial fat, were snap-frozen in liquid nitrogen and stored at − 80 °C, were provided by Dr. K.B. Marguiles (University of Pennsylvania). A piece of mouse or human tissue was weighed (20–100 mg) and cut into 2–3 mm^2^ pieces as described previously^41^. All the pieces were collected in a LoBind Eppendorf tube and IP lysis buffer (150 mM NaCl, 50 mM Tris/HCl pH 7.5, 1 mM EDTA, 0.5% Triton X-100, 0.5% NP-40, 1 mM DTT) with 0.5% SDS was added to make the concentration of 100 mg/mL. Approximately, 30–50 stainless steel beads (0.9–2.0 mm) were added to the mixture. Homogenization was then conducted twice using a Bullet Blender Gold homogenizer (Next Advance, Troy, NY) at a speed of 6 for 4 min. If white fibers were still present in the samples, the sample was further homogenized using probe sonication at strength of 5 for 30 pulses. Following centrifugation at 17720×* g* for 5 min, a portion of the supernatant (typically 200 μL) was withdrawn and mixed with IP lysis buffer (150 mM NaCl, 50 mM Tris/HCl pH 7.5, 1 mM EDTA, 0.5% Triton X-100, 0.5% NP-40, 1 mM DTT) supplemented with 1× complete protease cocktail.

### IP and digestion of frataxin

The protocol we described previously was used^27^ with minor modifications. Briefly, a portion of tissue homogenate (typically 200 μL) was mixed with (typically 800 μL) ice-cold IP-lysis buffer (50 mM Tris, pH 7.5, 150 mM NaCl, 0.5% Triton X-100, 0.5% NP-40, 1 mM DTT) supplemented with 1× complete protease cocktail, to which human mature SILAC-frataxin (81-210) (25 ng) with [^13^C_6_^15^N_2_]-lysine and [^13^C_6_^15^N_1_]-leucine residues was added^27^ as internal standard. 10% SDS was added and the mixture was heated at 95 °C for 10 min, followed by centrifugation at 17,720×*g* for 10 min. The supernatant was applied to an Amicon Ultracel-50 K filters. The filters were spun at 3600×*g*, 4 °C for 30 min. The flowthrough was mixed with 10 μg of mouse anti-frataxin mAb (Abcam ab113691) or 10 μg of rabbit anti-frataxin pAb (Abcam ab175402). After immunoprecipitation conducted by rotating the mixture at 4 °C overnight, the solution was transferred to another LoBind Eppendorf tube containing 1 mg of protein G magnetic beads and incubated with the beads at 4 °C overnight. The supernatant was then separated and discarded before 100 μL elution buffer (300 mM acetic acid and 10% acetonitrile) was added to the beads and shaken in 1000 × r.p.m. at room temperature for 1 h. The elution was separated from the magnetic beads, and dried under nitrogen flow. The samples were then dissolved in 25 mM aqueous NH_4_HCO_3_ solution (50 μL) containing 500 ng trypsin protease. Alternatively, the samples were dissolved in 50 mM ammonium acetate pH 4 containing 500 ng Glu-C V8 protease. The digestion was conducted at 37 °C overnight before UHPLC/PRM-MS analysis as described below.

### Tissue sub-cellular fractionation

A piece of frozen mouse liver sample (mouse information provided above) was homogenized and separated in into cytosolic, mitochondrial, and nuclear fractions using a modification of the method of Trefely et al.^31^ The tissue samples were weighed (approximately 30 mg) and cut into 2–3 mm^2^ pieces. All the pieces were collected in a LoBind Eppendorf tube containing 1 mL of MS buffer (210 mM mannitol, 70 mM sucrose, 5 mM Tri-HCl (pH 7.5) and 1 mM EDTA (pH 7.5). Liver tissue was homogenized using a 1 mL Corning Teflon pestle operated at 1600× r.p.m. and stroking the tissue suspension placed in a glass potter 10 times. During homogenization, the glass potter was in a beaker with ice. Both the pestle and glass potter were pre-chilled before homogenization. The homogenate was transferred to a 1.5 mL LoBind Eppendorf tube, and was centrifuged at 1300×*g* for 10 min at 4 °C to separate the nuclear and other debris. The supernatant was transferred without disturbing pellet (nuclei and debris) to another 1.5 mL LoBind tube, and was centrifuged at 10,000×*g* for 20 min at 4 °C to separate the mitochondria. The supernatant (cytosol) was transfer to another 1.5 mL LoBind tube without disturbing pellet (mitochondria). To the pellet (either nuclei or mitochondria) was added 1 mL IP lysis buffer 50 mM Tris, pH 7.5, 150 mM NaCl, 0.5% Triton X-100, 0.5% NP-40, 1 mM DTT) supplemented with 1× complete protease cocktail, to which 50 ng frataxin with isotopically labeled lysine and leucine was spiked as internal standard. To the cytosol was added 100 μL of 10× IP lysis buffer (500 mM Tris, pH 7.5, 1.5 M NaCl, 5% Triton X-100, 5% NP-40, 10 mM DTT) and 50 ng isotopically labeled frataxin was spiked as internal standard. A portion of each tissue fractionation (in IP lysis buffer before ISTD was spiked) was withdrawn for protein quantification and western blot analysis. IP and digestion were carried out in the same manner as described above.

### Protein quantification and western blot

Protein quantification was performed using a Bradford assay for each fraction of liver tissue (nuclei, mitochondria and cytosol) according to the manufacturer’s protocol. A series of BSA solutions in IP lysis buffer including 6 different concentration points (0.03, 0.06, 0.13, 0.19, 0.25, 0.38, 0.5 mg/mL) were used to create calibration curve. After determining the protein concentration, a portion from each fraction (~ 10 μg) was mixed with 5 μL NuPAGE LDS sample buffer (4X) containing 8% BME. The samples were then heated to 95 °C for 10 min before loading to 12% NuPAGE Bis–Tris protein gel. The gel was run under 150 V for 1.25 h until the blue dye ran to the bottom of the gel. The resulting gels were transferred onto either a 0.20 µm or 0.45 µm pore size nitrocellulose membranes according to manufacturer’s instructions (Invitrogen, Cat #s LC2000 and LC2006, respectively). The following rabbit Abs were then used for western blot analysis: anti-histone H3 pAb (1:1000, Invitrogen, cat # PA5-16183), anti-histone H4 pAb (1:1000, Invitrogen, cat # 16047-1-AP), anti-FASN mAb (1:1000, Invitrogen, cat # MA5-14887), and anti-VDAC mAb (1:1000, Cell Signaling Technology, cat # 4661S) for primary antibody incubations. Prior to incubation of the membrane with the anti-VDAC mAb, the membrane probed with anti-FASN mAb was stripped using a mild solution containing 200 mM glycine, 3.5 mM sodium dodecyl sulfate, and 10% Tween-20, pH 2.2. A goat anti-rabbit IgG (LI-COR, cat # 926-32211) was used as the secondary antibody. An Odyssey CLx imaging system (Thermo Fisher) with Image Studio v2.0.38 software (LI-COR Biosciences) was used to acquire images which were exported as TIFF files and annotated using Adobe Illustrator software v24.2.

### 2D-nano-UHPLC-PRM/MS

MS was conducted using a high-resolution Q-Exactive HF hybrid quadrupole-orbitrap mass spectrometer coupled to a Dionex Ultimate 3000 RSLCnano with capillary flowmeter chromatographic systems (Thermo Fisher Scientific, San Jose, CA, USA) as described previously with minor modifications^27^. The 2D system was setup in a pre-concentration mode which was composed of a ten-port valve, one nanopump for delivering solvents to analytical column, and a micropump for delivering solvents to trapping column. The 2D nano-UHPLC system was controlled by Xcalibur software from the Q-Exactive mass spectrometer. The UHPLC trapping column was an Acclaim PepMap C_18_ cartridge (0.3 mm × 5 mm, 100 Å, Thermo Scientific) and the analytical column was a C_18_ AQ nano-UHPLC column with a 10 µm pulled tip (75 µm × 25 cm, 3 µm particle size; Columntip, New Haven, CT). Samples (8 μL) were injected using the microliter-pickup injection mode. Loading solvent was water/acetonitrile (99.7:0.3; v/v) containing 0.2% formic acid. In the sample loading step, the valve stayed in loading position (1–2) with loading solvent at 10 μL/min for 3 min. In the elution and analysis step, the valve stayed in injection position (1–10) at which the trapping column was connected to the nanopump and the analytical column, and samples were back-flushed into the analytical column. Washing of the trapping column using the nanopump was continued until 5 min before the end of the run. Samples were eluted in the mass spectrometer with a linear gradient at a flow rate of 0.4 μL/min. Solvent A was water/acetonitrile (99.5:0.5; v/v) containing 0.1% formic acid, and solvent B was acetonitrile/water (98:2, v/v) containing 0.1% formic acid. The gradient on the analytical column was as follows: 2% B at the start, 5% B at 9 min, 27% B at 27 min, 95% B at 31 min, held for 14 min, then re-equilibrated at 2% B from 48 to 60 min. Ionization was conducted using a Nanospray Flex ion source (Thermo Scientific). Mass spectrometer operating conditions were as follows: spray voltage 2800 V, ion transfer capillary temperature 275 °C, ion polarity positive, S-lens RF level 55, in-source CID 1.0 eV. Both full scan MS/MS and PRM/MS were used. The full scan MS/MS parameters were: resolution 120,000, AGC target 3e6, maximum IT 100 ms, scan range *m/z* 400–1300. The PRM/MS parameters were: resolution 60,000, AGC target 2e5, maximum IT 80 ms, loop count 5, isolation window 2.0 Da, NCE 25.

### Data analysis

Data analysis for protein quantification was performed using Skyline (MacCoss Laboratory, University of Washington, Seattle, WA). The peak area ratio of each PRM/MS transition for each unlabeled/light (L) peptide to labeled/heavy (H) peptide was calculated by the Skyline software and used for absolute quantification. The N-terminal peptide ratios for the mouse proteins were calculated by the average L/H ratios of the most intense PRM/MS transition to the [M + 3H]^3+^ (m/z 611.964) to y_11_^+^ (1274.5948-1274.5998) PRM/MS transition for [^13^C^15^N]-S^81^GT**L**GHPGS**L**DETTYER^98^ (Figs. 3 and 4). Concentrations of mouse mature frataxin proteins were determined from the L/H ratio of each N-terminal peptide to the [^13^C^15^N]-SGT peptide from the 25 ng human mature SILAC-frataxin (81-210) that was added. For human mature frataxin (81-210), concentrations in heart tissue were determined from the L/H ratios of the three most intense PRM/MS transitions from each of three peptides (S^81^GTLGHPGSLDETTYER^97^, L^136^GGDLGTYVINK^147^, and Q^150^IWLSSPSSGPK^161^, Fig. 6A–C) and interpolation from the relevant standard curves as described previously^27^.

### Statements

Mouse tissues: All protocols and surgical experiments on mice were performed in accordance with and approved by the Institutional Animal Care and Use Committee (IACUC) of the University of Pennsylvania. Human tissues: The method for acquisition of human hearts from organ donors undertaken under NIH grant RO1HL105993 (Marguiles, K.B.; PI), was approved by the University of Pennsylvania Hospital Institutional Review Board. Prospective written informed consent in accordance with the Declaration of Helsinki and the approved guidelines of the University of Pennsylvania for research on the use of heart tissue, was obtained from next-of-kin of organ donors.

## Supplementary information

Supplementary Information 1.
