# Supplementary material for: Extra-mitochondrial mouse frataxin and its implications for mouse models of Friedreich’s ataxia
Source: Sci Rep. 2020 Sep 25;10:15788. doi: 10.1038/s41598-020-72884-w (PMC7519113; doi:10.1038/s41598-020-72884-w)
Supplement: Supplementary file 1 — Supplementary Information 1. [file 41598_2020_72884_MOESM1_ESM.pdf]

Supplementary Information

Extra-mitochondrial mouse frataxin and its implications for mouse models of Friedreich's ataxia

Liwei Weng,[†‡§] Laurent Laboureur,[†‡§] Qingqing Wang,[†‡] Lili Guo,[†‡] Peining Xu,[†‡] Leah Gottlieb,[†‡] David R. Lynch,[†¶] Clementina Mesaros,[†‡] and Ian A. Blair[*†‡]

[†]Penn Medicine/CHOP Center of Excellence in Friedreich's Ataxia, University of Pennsylvania, Philadelphia, Pennsylvania 19104, United States

[‡]Center of Excellence in Environmental Toxicology Center, Department of Systems Pharmacology and Translational Therapeutics, Perelman School of Medicine, University of Pennsylvania, Philadelphia, Pennsylvania 19104, United States

[¶]Departments of Pediatrics and Neurology, Perelman School of Medicine, University of Pennsylvania, Philadelphia, Pennsylvania 19104, United States

[§]Authors made an equal contribution

*Corresponding author: Tel: +1-215-573-9880. Fax: +1-215-573-9889. E-mail: ianblair@upenn.edu.

ORCID
Ian A. Blair: 0000-0003-0366-8658

Supplementary Figures

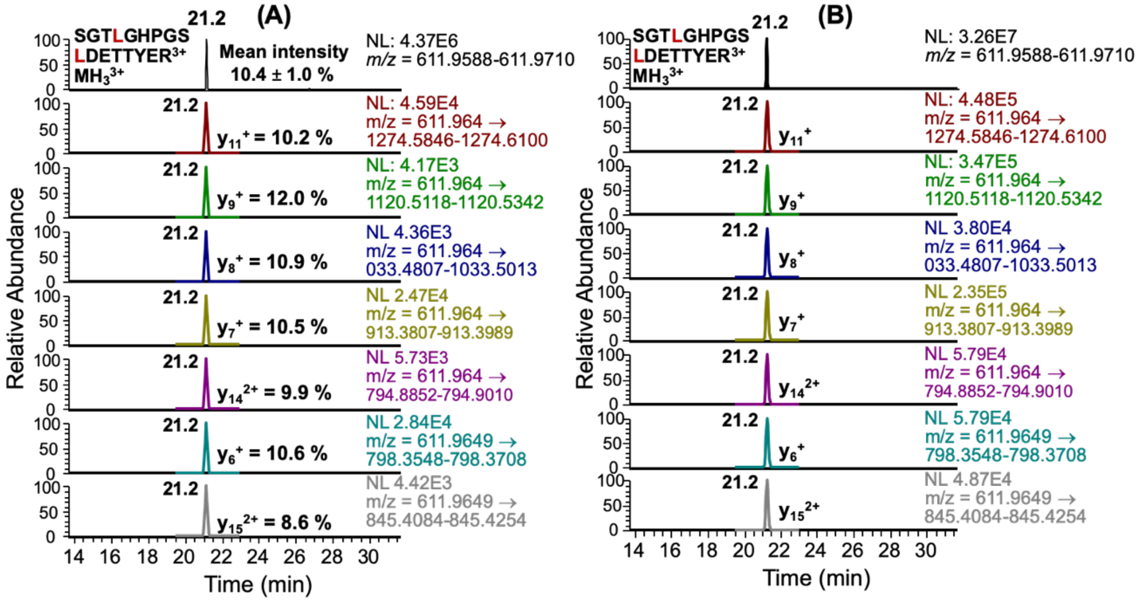

Supplementary Fig. 1: UHPLC-PRM/MS analysis of the N-terminal peptide from human mature SILAC frataxin isolated by IP using two different antibodies non-covalently bound to protein G magnetic beads. (A) Abcam anti-frataxin rabbit pAb 175402. (B) Abcam anti-frataxin mouse mAb 113691. The recovery determined from mean of the ratios of the major PRM signals for S^{78}GT**L**GHPGS**L**DETTYER^{79} from human SILAC-mature frataxin (81-210) isolated using rabbit pAb 175402 was 10 % compared with S^{78}GT**L**GHPGS**L**DETTYER^{79} from human SILAC-mature frataxin (81-210) isolated using mouse mAb 113691. **L**= [$^{13}C_6{}^{15}N_1$]-leucine.

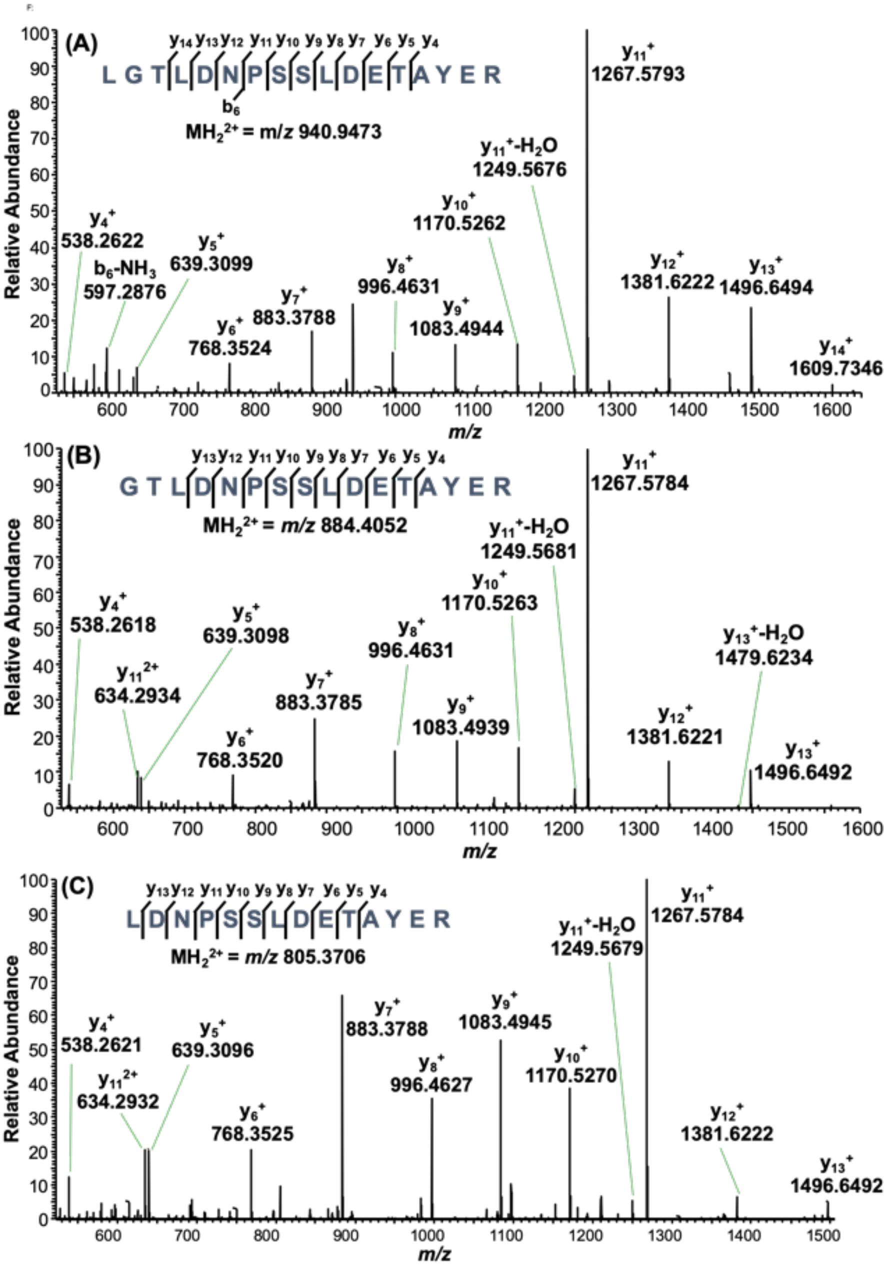

Supplementary Fig. 2: Product ion spectra of N-terminal tryptic peptides from mouse mature frataxin isolated from mouse liver. (A) L^{78}GTLDNPSSLDETAYER^{94}. (B) G^{79}TLDNPSSLDETAYER^{94} (C). L^{81}DNPSSLDETAYER^{94}.

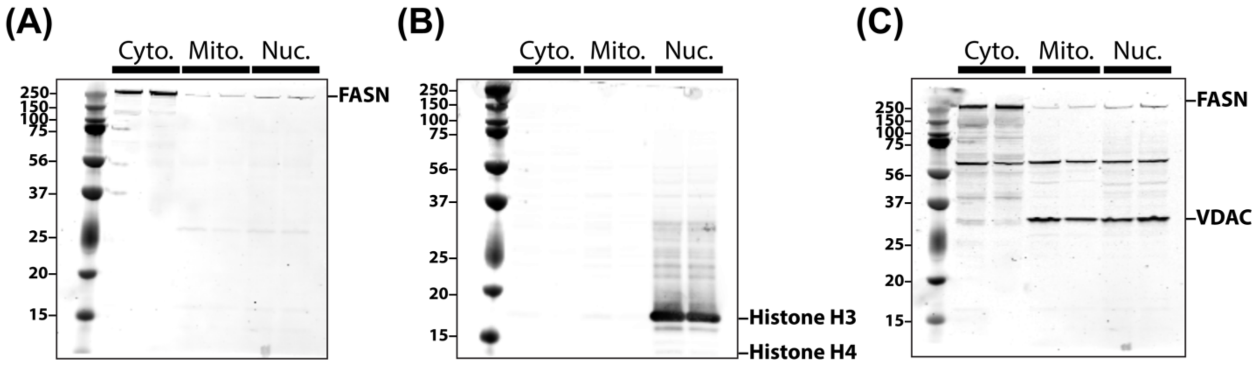

Supplementary Fig. 3: Markers of sub-cellular fractionation of mouse liver tissues (n=2) visualized by western blot with cytoplasmic, mitochondrial, and nuclear fractions indicated. (A) FASN cytosolic protein (200 kDa). (B) Histone H3 and H4 (17 kDa and 11 kDa respectively). (C) VDAC mitochondrial membrane protein (31 kDa) using the stripped blot shown in Panel (A).

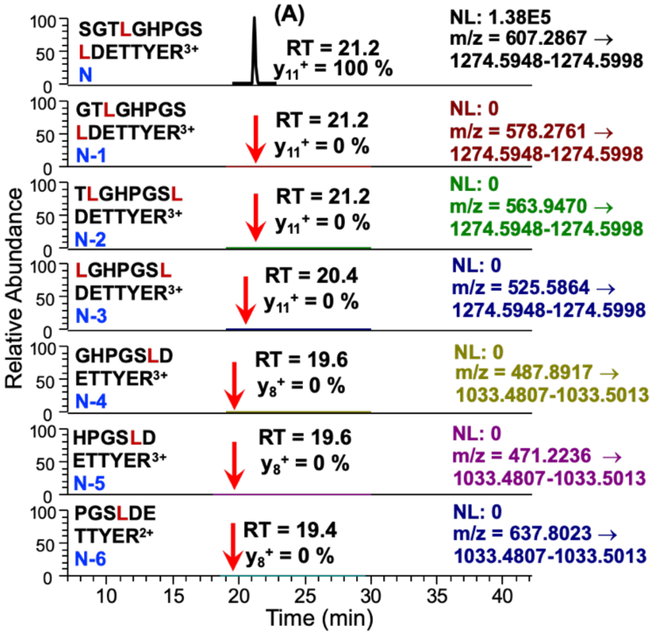

Supplementary Fig. 4: UHPLC-PRM/HRMS analysis of potential truncated peptides from human SILAC-frataxin internal standard (25 ng) added to mouse heart tissue. Red arrows denote the retention times of authentic peptide standards. **L**= [$^{13}C_6{}^{15}N_1$]-leucine.

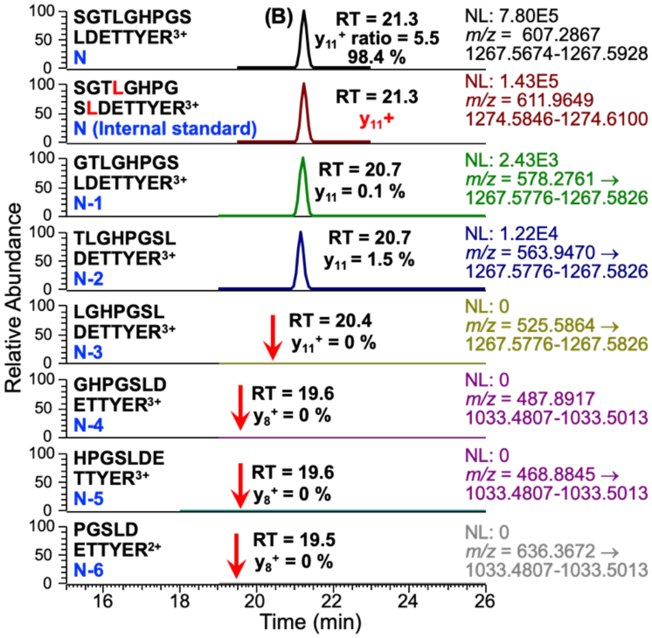

Supplementary Fig. 5: UHPLC-PRM/MS analysis of potential truncated peptides from human frataxin. Human SILAC-frataxin internal standard (25 ng) added to mouse heart tissue. Red arrows denote the retention times of authentic peptide standards. **L**= [$^{13}C_6{}^{15}N_1$]-leucine.

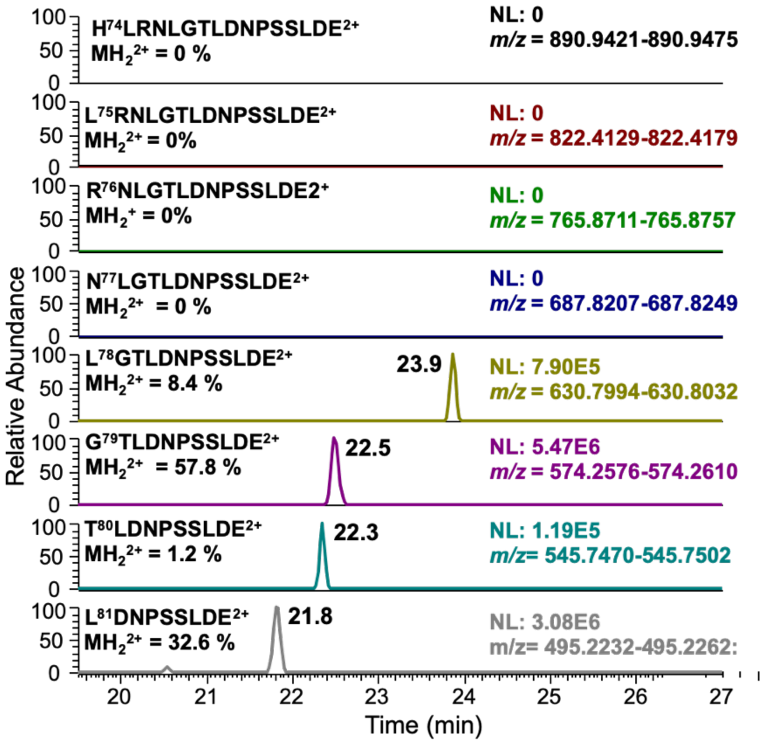

Supplementary Fig. 6: UHPLC-PRM/MS analysis of potential N-terminal Glu-C peptides from mouse liver frataxin.
